# Case Report: Lenalidomide as a Second-Line Treatment for Bortezomib-Ineffective Nephrotic Syndrome Caused by LCDD: 2 Case Reports and a Literature Review

**DOI:** 10.3389/fmed.2021.706971

**Published:** 2021-10-08

**Authors:** Xin Zhang, Xiao-juan Yu, Su-xia Wang, Fu-de Zhou, Ming-hui Zhao

**Affiliations:** ^1^Renal Division, Department of Medicine, Peking University First Hospital, Beijing, China; ^2^Institute of Nephrology, Peking University, Beijing, China; ^3^Renal Pathology Center, Institute of Nephrology, Peking University First Hospital, Beijing, China; ^4^Key Laboratory of Renal Disease, Ministry of Health of China, Beijing, China; ^5^Key Laboratory of Chronic Kidney Disease Prevention and Treatment, Ministry of Education of China, Beijing, China; ^6^Research Units of Diagnosis and Treatment of Immune-Mediated Kidney Diseases, Chinese Academy of Medical Sciences, Beijing, China; ^7^Laboratory of Electron Microscopy, Pathological Centre, Peking University First Hospital, Beijing, China; ^8^Peking-Tsinghua Center for Life Science, Beijing, China

**Keywords:** case report, light-chain deposition disease, nephrotic syndrome (NS), lenalidomide, monoclonal gammopathies of renal significance

## Abstract

**Background:** Light-chain deposition disease (LCDD) is a rare systemic disorder characterized by the deposition of monoclonal light chains in organs. The kidney is a prominent target of light-chain deposition, with a median time to end-stage renal disease (ESRD) of 2.7 years and 5-year ESRD-free survival of 37%. The therapeutic management of LCDD remains ill-defined. In addition to bortezomib-based therapy as first-line therapy, the effect of lenalidomide on LCDD is rarely reported.

**Case Presentation:** This study describes two male LCDD patients in their 60s with nephrotic syndrome and moderately impaired renal function. One patient had monoclonal IgGλ with underlying MGRS, and another had monoclonal IgGκ with underlying monoclonal gammopathy that developed into symptomatic MM during follow-up. The hallmarks of this disease were consistent with previous reports. Both patients initially received BCD therapy, but no hematological response was observed. Consequently, the nephrotic syndrome was refractory. Sequential Rd therapy was initiated, and partial hematological response and nephrotic remission were observed in the IgGλ patient but absent in the IgGκ patient.

**Conclusion:** Limited reports have demonstrated the effect of lenalidomide in LCDD. We report the outcome of lenalidomide in two cases of bortezomib-resistant LCDD. This treatment might be a beneficial supplement for those unresponsive or intolerant to bortezomib in LCDD, but the effect should be prospectively investigated.

## Background

Light-chain deposition disease (LCDD) is a rare systemic disorder characterized by the deposition of monoclonal light chains in organs (1). The kidney is a prominent target of light-chain deposition, but the liver, lung, and gastrointestinal tract are also involved (2, 3). As an abnormal clone of B cells overproduces free light chains, LCDD is typically described in the course of plasma cell dyscrasias, ranging from malignant multiple myeloma (MM) to relatively benign monoclonal gammopathy of undetermined significance (MGUS) (4). Renal involvement of LCDD typically presents with nephrotic syndrome or asymptomatic proteinuria accompanied by microhaematuria and impaired renal function. Light microscopy of kidney biopsies often reveals mesangial proliferation, mesangial matrix expansion, nodular glomerulosclerosis, and tubular atrophy. Immunohistochemical staining was positive for light chains (mostly κ, rarely λ) along the glomerular basement membrane (GBM) or tubular basement membrane (TBM). Furthermore, Congo-red staining is negative (4–7). It is recognized that the renal prognosis of LCDD is poor, with a median time to end-stage renal disease (ESRD) of 2.7 years and 5-year ESRD-free survival of 37% (5). The therapeutic management of LCDD remains ill-defined, and the treatment recommendations are similar to those of MM (8). Recent studies have suggested that the combined administration of bortezomib, cyclophosphamide, and dexamethasone (BCD) could represent a successful first-line therapy (9–11). However, some patients are resistant to BCD therapy. Given that lenalidomide, an immunomodulatory drug, is an effective and well-tolerated regimen to treat multiple myeloma (MM) patients relapsing after bortezomib induction therapy (12), lenalidomide-based therapy might therefore improve the prognosis of LCDD-induced nephrotic syndrome (13, 14). Here, we report lenalidomide-based therapy in 2 cases of LCDD-induced nephrotic syndrome with a poor response to BCD therapy and conduct a literature review.

## Case Presentation

### Case 1

**Figure 1:**
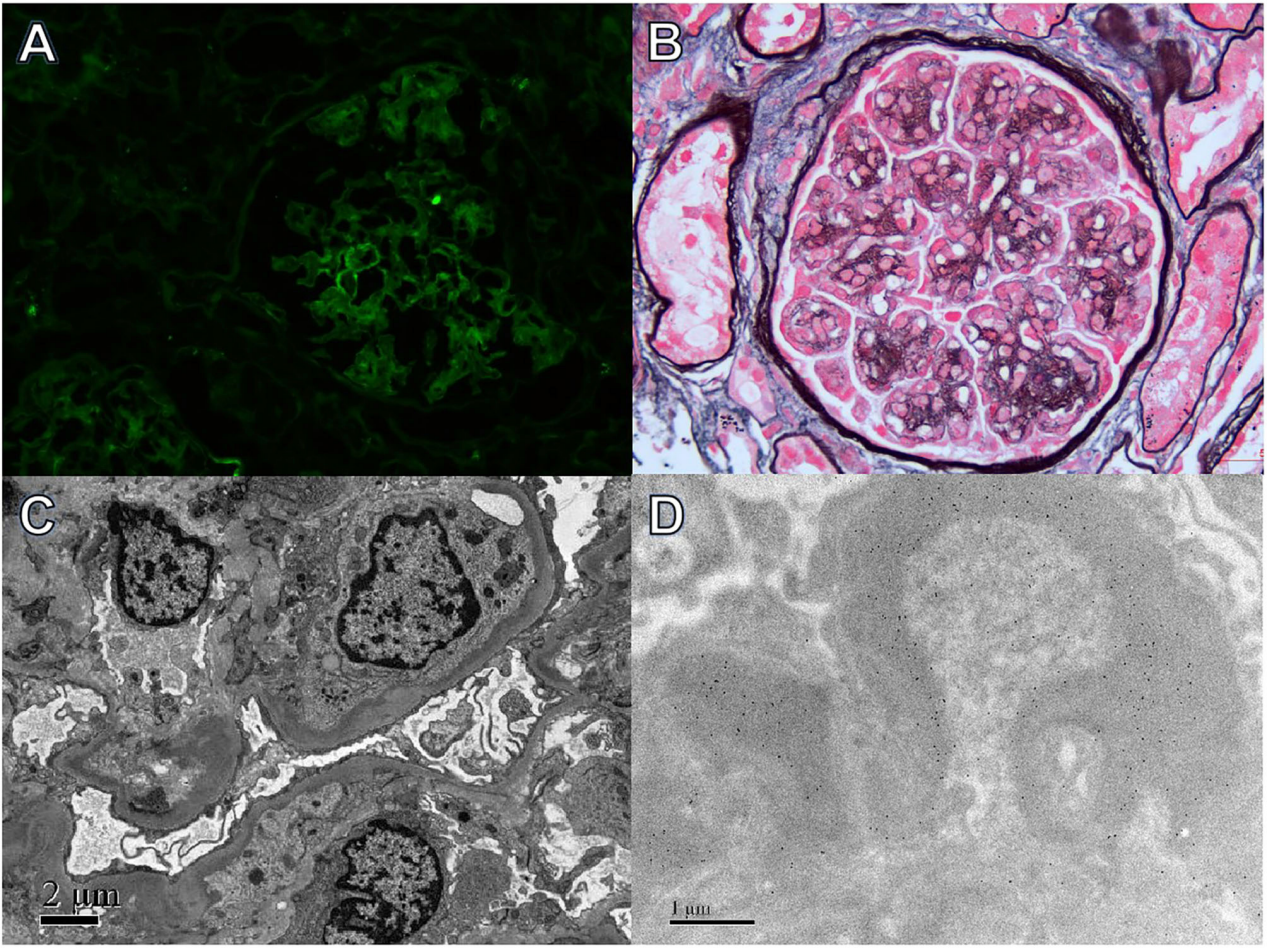
Light, immunofluorescence, electron, and immune-electron microscopy findings in Case 1. **(A)** Immunofluorescence studies revealed bright λ staining in the segmental mesangial area and along capillary walls (×400). **(B)** Light microscopy showed a mesangial proliferative pattern of injury (periodic acid-silver methenamine + Masson trichrome staining, ×400). **(C)** Electron microscopy revealed electron-dense deposits in segmental mesangial and subendothelial regions (×800). **(D)** Immunoelectron microscopy demonstrated λ deposition in the mesangial area and segmental subendothelial regions (×8,000).

**Figure 2:**
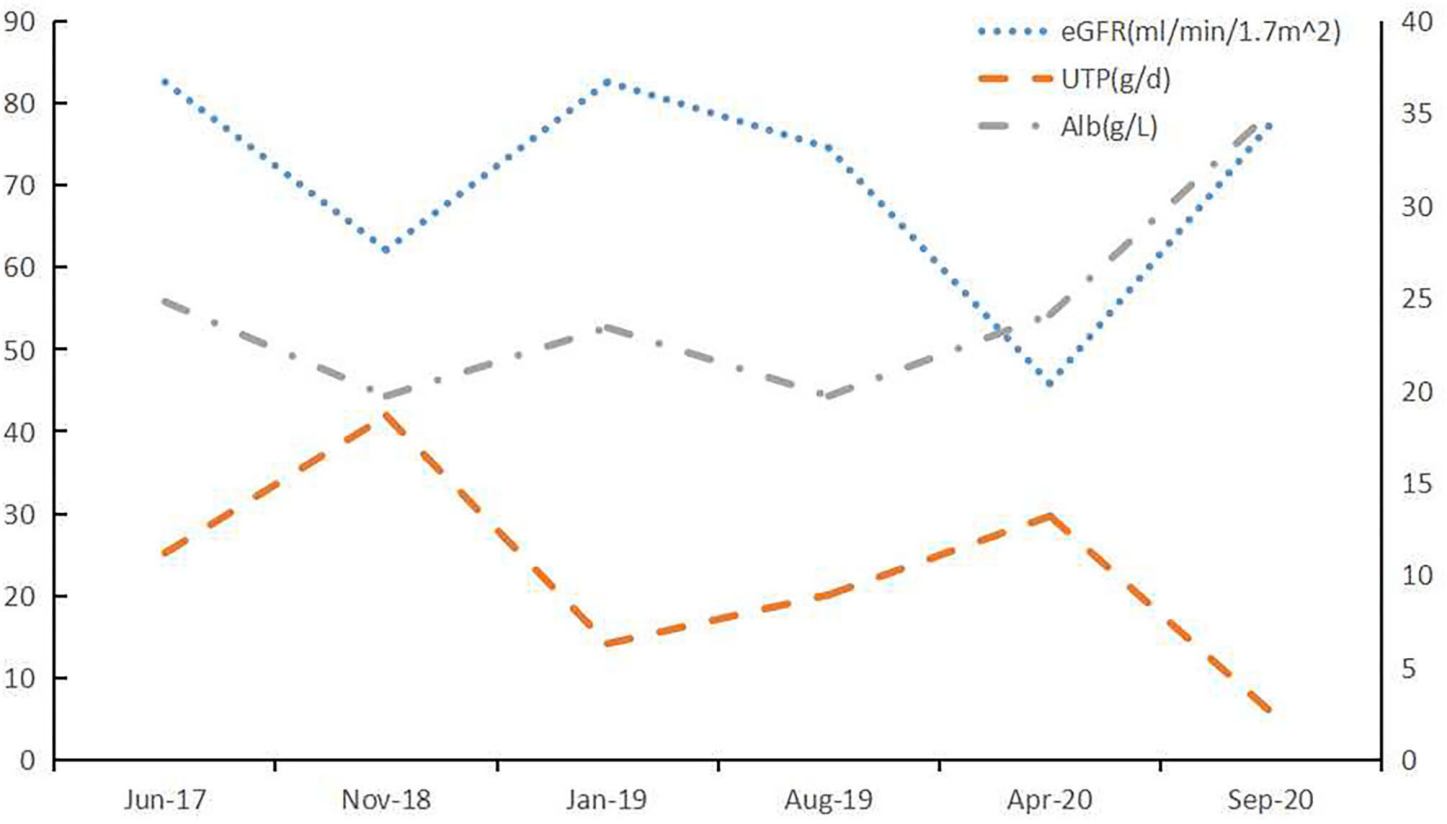
Light, immunofluorescence, electron, and immunoelectron microscopy findings in Case 2. **(A)** Immunofluorescence staining revealed bright κ deposition in the mesangial area and along capillary walls (×400). **(B)** Light microscopy revealed a membranoproliferative pattern of injury (periodic acid-silver methenamine + Masson trichrome staining, ×400). **(C)** Electron microscopy demonstrated electron-dense deposits in subendothelial and mesangial regions (×8,000). **(D)** Immunoelectron microscopy showed κ deposition in the mesangial area and inner GBM (×8,000).

A 60-year-old male visited our hospital with proteinuria for 13 years and lower limb edema for 2 years. His initial proteinuria was 11.2 g/d, serum albumin was 24.8 g/L, and eGFR was 82.563 ml/min/1.73 m^2^, leading to a diagnosis of nephrotic syndrome. He once received conservative therapy, including RASis, for more than 1 year, but the nephrotic syndrome did not achieve remission. When he presented to our hospital, he had a normal vital sign with severe edema of the lower extremities. He had a past medical history of HBV infection, hypertension, coronary heart disease, and coronary artery bypass grafting (CABG). His laboratory results indicated nephrotic syndrome (proteinuria 18.63 g/d and albumin 19.8 g/L), synchronous microscopic haematuria (200–300 erythrocytes/HPF), and worsening renal function (eGFR 62.118 ml/min/1.73 m^2^). No anemia, hypercalcemia, or myocardial injury was detected. He had a normal IgG serum level with lower serum levels of IgA and IgM (12.9, 0.53, and 0.57 g/L, respectively) and a lower C3 serum level (0.426 g/L) with normal C4 (0.151 g/L). His complement factor H, anti-CFH antibody, and ADAMTs13 activity were normal. He had monoclonal IgGλ on serum and urine immunoelectrophoresis. The serum free light chain ratio was abnormal (κ/λ 0.0875, κ 30.4 mg/L, λ 347.5 mg/L). Bone marrow aspiration revealed no abnormal plasma cells. He received a renal biopsy, and light microscopy revealed 48 glomeruli with 15 ischaemic scleroses. The remaining glomeruli exhibited moderate to severe diffuse mesangial and matrix hyperplasia, diffuse endocapillary hyperplasia, and focal segmental nodular sclerosis. Thickening of GBM and atrophy of tubules were also observed. Congo red staining was negative. Immunofluorescence results revealed prominent λ deposition alongside the glomerular capillary wall and segmental mesangial region. Electron microscopy revealed electron-dense deposits in the subendothelial area and along segmental GBM, which were prominent λ under immunoelectron microscopy (Figures 1A–D). The treatment was started with bortezomib (2 mg, once weekly), cyclophosphamide (200 mg, once weekly), and dexamethasone (20 mg, once weekly) (BCD therapy). Oral entecavir was administered for the prevention of HBV reactivation. After six cycles of BCD treatment, his hematological response did not achieve remission (κ/λ 0.137, κ 32.0 mg/L, λ 233.3 mg/L), and his nephrotic syndrome was not relieved (UTP 13.2 g/d, Alb 24.1 g/L, eGFR 45.84 ml/min/1.7 m^2^). After consulting the hematology physician, his treatment was changed to lenalidomide (25 mg daily, days 1 through 21 of each 28-day cycle) and dexamethasone (20 mg once weekly) (Rd therapy). After eight cycles of Rd therapy, his hematological response achieved partial remission (κ/λ ratio 0.28, κ 35.4 mg/L, λ 125 mg/L), his nephrotic syndrome achieved partial remission (UTP 2.7 g/d, Alb 35.1 g/L), and impaired renal function was restored (eGFR 77.541 ml/min/1.7 m^2^) (Figure 2).

### Case 2

**Figure 3:**
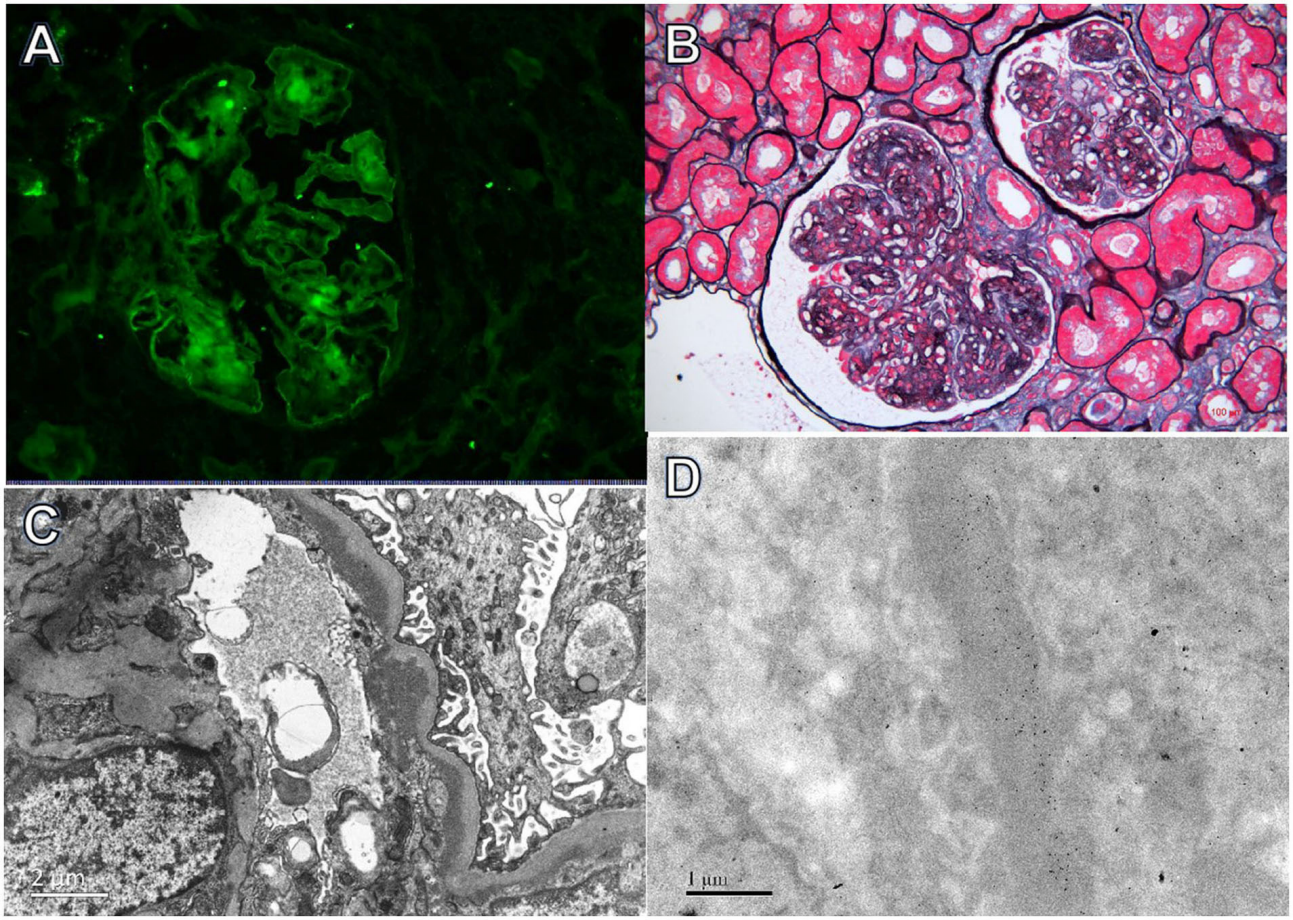
Clinical course of Case 1. After treatment with lenalidomide, the renal impairment and proteinuria caused by LCDD was improved. BCD: bortezomib (2 mg, once weekly), cyclophosphamide (200 mg, once weekly), and dexamethasone (20 mg, once weekly); Rd: lenalidomide (25 mg daily, on Days 1 through 21 of each 28-day cycle) and dexamethasone (20 mg once weekly).

**Figure 4:**
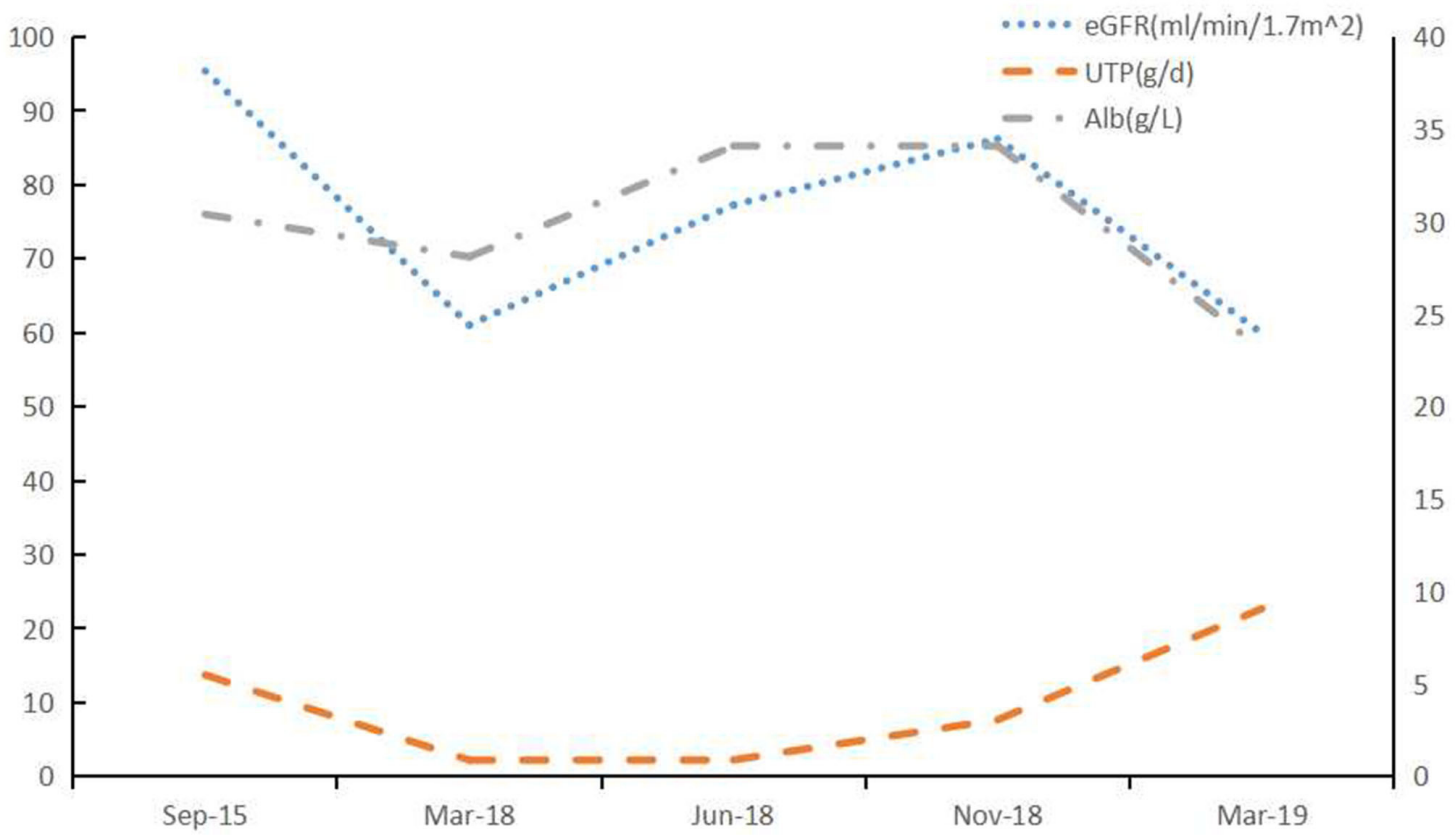
Clinical course of Case 2. After treatment with lenalidomide, the renal impairment and proteinuria caused by LCDD was aggravting. BCD: bortezomib (2 mg, once weekly), cyclophosphamide (200 mg, once weekly), and dexamethasone (20 mg, once weekly); Rd: lenalidomide (25 mg daily, on Days 1 through 21 of each 28-day cycle) and dexamethasone (20 mg once weekly).

A 63-year-old male who had proteinuria for three years presented at our department for aggravating anemia and impaired renal function. Before he came to our hospital, he had already received a renal biopsy which revealed an MPGN-like glomerular injury. He also had monoclonal IgGκ on serum IFE. He had received a 1-year course of immunosuppressive therapy combined with prednisone (15 mg daily) and tacrolimus (1.5 mg twice daily) due to nephrotic syndrome (UTP 5.48 g/d, Alb 30.4 g/L, eGFR 95.372 ml/min/1.7 m^2^). After immunosuppressive therapy, his nephrotic syndrome achieved complete remission, but his anemia and impaired renal function persisted. His vital signs were normal when referred to our hospital. His past medical history included hypertension and dyslipidemia. The laboratory findings indicated relieved proteinuria but persistent hypoproteinaemia and impaired renal function (UTP 0.88 g/d, Alb 28.6 g/L, eGFR 61.022 ml/min/1.7 m^2^). The hematological evaluation revealed moderate anemia (Hgb 89 g/L). He had no microscopic haematuria, hypercalcemia, or myocardial injury. He had normal IgG and IgA serum levels with a lower serum level of IgM (0.39 g/L) and a deficient C4 serum level (<0.07 g/L) with a relatively low C3 level (0.67 g/L). Complement factor H, anti-CFH antibody, and ADAMTs13 activity were normal. He had monoclonal IgGκ+κ on serum IFE, and monoclonal κ on urine IFE. The serum free κ/λ ratio was 43.7 (κ 1580.0 mg/L, λ 36.1 mg/L). He also had cryoglobulinemia, which was monoclonal IgG and κ, but without correlative clinical symptoms. The bone marrow aspiration revealed 4% plasma cells with 2.5% naive plasma cells, while the bone marrow biopsy did not reveal evidence of plasmacytoma. A repeated renal biopsy was conducted. Light microscopy showed moderate diffuse mesangial cell proliferation and an increase in the mesangial matrix that contained nodular lesions and a thickening and double contour of the GBM. The tubulointerstitium exhibited multifocal lymphocyte and monocyte infiltration, fibrosis, and moderate tubular atrophy. No microthrombus was observed. Congo red staining was negative. Immunofluorescence results were strongly positive for κ light-chain deposition along the glomerular capillary wall, mesangial region, and segmental TBM. Electron microscopy revealed electron-dense deposits on the inner side of the GBM, mesangial area, and endolysosome of renal tubular epithelial cells, which were prominent κ under immunoelectron microscopy (Figures 3A–D). Given his outstanding anemia, the diagnosis of multiple myeloma was suspected but unproven. Therefore, he was temporally diagnosed with monoclonal gammopathy of renal significance. A combination of BCD therapy was administered (bortezomib 2 mg, once weekly, cyclophosphamide 200 mg, once weekly, and dexamethasone 20 mg, once weekly). After six cycles of BCD therapy, the status of anemia was not improved. However, proteinuria was exacerbated (UTP 3.04 g/d), and edema relapsed. He received repeated serum-free light chain tests and exhibited hematological progression (κ/λ ratio 168.1, κ 1697.5 mg/L, λ 10.1 mg/L,). His repeated bone marrow smear revealed 4.5% plasma cells, but plasmacytoma was observed in bone marrow biopsy. A diagnosis of MM was determined. He was referred to the haematologic department. The treatment was changed to Rd therapy (lenalidomide, 25 mg daily, days 1 through 21 of each 28-day cycle, and dexamethasone, 20 mg once weekly). However, the patient was intolerant to lenalidomide, presenting with severe skin rash. Thus, the lenalidomide dosage was decreased to 12.5 mg daily. After four courses of Rd therapy, his free light chain ratio showed improvement (κ 1185 mg/L, λ 42.4 mg/L, κ/λ ratio 27.9), but his nephrotic syndrome and anemia both aggregated (UTP 9.07 g/d, Alb 23.1 g/L, and Hgb 49 g/L) with relatively preserved renal function (eGFR 59.911 ml/min/1.73 m^2^) (Figure 4). Then, he received treatment in another hospital, and the follow-up was conducted by telephone. He received IRd therapy (ixazomib, lenalidomide, dexamethasone) for an additional 5 months without a clear hematological or renal response, and the free κ value once reached ~5,000 mg/L. Thereafter, antiCD38 monoclonal antibody therapy was administered. His nephrotic syndrome achieved partial remission with stable eGFR, and free light-chain κ decreased to ~700 mg/L after 17 cycles of CD38 monoclonal antibody therapy. ASCT was considered as a further treatment option.

## Discussion and Conclusions

We reported two male LCDD patients in their 60s with nephrotic syndrome and moderately impaired renal function. One patient had monoclonal IgGλ with underlying MGRS, and the other had monoclonal IgGκ with underlying symptomatic MM. Both patients failed to respond to the initial BCD therapy. Sequential Rd therapy was initiated, and partial hematological response and nephrotic remission were observed in the IgGλ patient but absent in the IgGκ patient.

Given the rarity of LCDD, to date, there were only a few reports regarding the treatment of LCDD with lenalidomide. We reviewed the literature and identified 6 cases describing the effects of lenalidomide on LCDD. All of these studies were case reports (13–16) (Table 1). Among the six patients, four received bortezomib-based therapy before lenalidomide regimens. Patients #1 and #2 responded to BD therapy, but they were intolerant to bortezomib and withdrew from BD therapy due to severe neurotoxicity(#1) and GI toxicity(#2). After lenalidomide therapy, 5 out of 6 patients reached VGPR in hematological status, and all experienced renal remission (CR or PR). No treatment-associated side effects were reported. But one patient in our report presented with severe skin rash induced by lenalidomide, leading to dosage adjustment.

**Table 1:** Reported cases of LCDD treated with lenalidomide.

	**No**.	**Patient Age/Gender**	**MM**	**NS**	**Renal function**	**External involved organs**	**Light chain isotype**	**Treatment**	**Hematological response**	**Organ response**
Gkotzamanidou et al. (15)	1	69/M	–	+	CKD 4	None	λ	Predisome+CTX 18 months → BD 5 cycles → RdC 15 cycles	VGPR	Partial renal remission
Jimenez-Zepeda et al. (16)	2	61/M	–	–	ESRD	GI tract, heart and liver	κ	Bortezomib 4 times → Rd 10 cycles	PR	Persistent ESRD GI symptoms resolved Stable cardiac function
Kimura et al. (13)	3	69/M	–	–	CKD 3b	None	κ	BD 4 cycles → Rd 30^+^ cycles	VGPR	Complete renal remission
	4	59/M	–	+	CKD 3a	None	κ	Rd 30 cycles	VGPR	Complete renal remission
	5	60/F	–	–	CKD 3a	None	κ	Rd 5 cycles → ASCT → low dose Rd 10 cycles	VGPR	Complete renal remission
Mima et al. (14)	6	80/F	+	+	CKD 3b	None	λ	BCD 4 cycles → Rd 4 cycles	VGPR	Partial renal remission

*LCDD, Light chain deposition disease; MM, multiple myeloma; NS, nephrotic syndrome; F, females; M, males; GI, gastrointestinal; ASCT, autologous peripheralblood stem cell transplantation; ESRD, end stage of renal disease; CTX, cyclophosphamide; BD, bortezomib and dexamethasome; BCD, bortezomibn, dexamethasome, and cyclophosphamide; Rd, lenalidomide and dexamethasome; RdC, lenalidomide, dexamethasome and cyclophosphamide; PR, partial response; VGPR, very good partial response*.

Bortezomib-based chemotherapy prolonged the renal survival of LCDD through high hematological responses; more than 90% of the patients showed at least a partial response in hematological status, and ~50% exhibited a renal response (9). However, some patients are resistant to bortezomib therapy. The underlying mechanisms are not yet evident in LCDD. But referring to the studies in MM, the potential mechanisms have been suggested as follows: proteasome mutations, such as proteasome β5 subunit (PSMB5); key stress signal pathway cross-talk, such as HSP family members; and bone marrow microenvironment changes, such as overexpression of IGF-1 (17). Interestingly, in light-chain amyloidosis, another aberrant light-chain gammopathy, the presence of *t*_(11;14)_ is a marker of poor response to a bortezomib-based regimen (18, 19). We did not conduct a gene test or FISH test on bone marrow, so the resistance mechanism of our two patients remained undetermined. Lenalidomide, as an immunomodulatory drug, is introduced as a treatment option for bortezomib-resistant patients. This drug exhibits a broad mechanism of action that can be direct or indirect, including antiangiogenic, proapoptotic, antiproliferative, and immunomodulatory effects (20). In LCDD, the interaction of mesangial cells with monoclonal light chains leads to the activation of cytokines, such as TGF-β and VEGF (induced by NF-κB), in combination with the high production of matrix and extracellular matrix proteins that comprise the glomerular lesions in LCDD (21). Lenalidomide downregulates the NF-κB signaling pathway by activating monocytes while simultaneously upregulating IL-2 and interferon-γ production, which promotes the activation of T and natural killer cells and triggers apoptosis in immunoglobulin-secreting plasma cells (22, 23). In addition, the NF-κB signaling pathway might also act directly in mesangial cells to downregulate the production of cytokines, including TGF-β, and promote the repair of glomerular lesions, which needs further confirmation.

In a recently published case report, the anti-CD38 monoclonal antibody therapy resulted in a high hematological rate and preserved kidney function in 8 patients with refractory multiple myeloma-related LCDD (24). In our report, patient#2 showed a hematological response to the anti-CD38 monoclonal antibody therapy after the failure of lenalidomide-based treatment, indicating the promising future of anti-CD38 monoclonal antibody in LCDD.

We report using the lenalidomide regimen in two cases of LCDD-induced nephrotic syndrome who failed to respond to bortezomib therapy. This treatment might be a beneficial supplement for those unresponsive or intolerant to bortezomib in LCDD, but the effect should be prospectively investigated.

## Data Availability Statement

The raw data supporting the conclusions of this article will be made available by the authors, without undue reservation.

## Ethics Statement

The studies involving human participants were reviewed and approved by the Ethics Committee of the Peking University First Hospital. The patients/participants provided their written informed consent to participate in this study.

## Author Contributions

XZ, X-jY, and M-hZ analyzed and interpreted the patient clinical data. XZ and X-jY performed the literature review and were major contributors in writing the manuscript. S-xW and X-jY performed the histological examination of the kidney biopsy. XZ, F-dZ, and X-jY followed up with the patient and collected the clinical data. All authors read and approved the final manuscript.

## Funding

This study was supported by grants from the National Natural Science Foundation of China (Nos. 81470956 and 81500543) and CAMS Innovation Fund for Medical Sciences (2019-I2M-5-046).

## Conflict of Interest

The authors declare that the research was conducted in the absence of any commercial or financial relationships that could be construed as a potential conflict of interest.

## Publisher's Note

All claims expressed in this article are solely those of the authors and do not necessarily represent those of their affiliated organizations, or those of the publisher, the editors and the reviewers. Any product that may be evaluated in this article, or claim that may be made by its manufacturer, is not guaranteed or endorsed by the publisher.

## Abbreviations

ASCT: autologous peripheral blood stem cell transplantation
BD: bortezomib and dexamethasone
BCD, bortezomib, dexamethasone: and cyclophosphamide
CR: complete response
CTX: cyclophosphamide
ESRD: end-stage of renal disease
FISH: fluorescence *in situ* hybridization
LCDD: light-chain deposition disease
MM: multiple myeloma
MGRS: monoclonal gammopathy of renal significance
MPGN: membranous proliferative glomerulonephritis
NS: nephrotic syndrome
PR: partial response
RASi: renin-angiotensin-aldosterone system inhibitor
Rd: lenalidomide and dexamethasone
RdC, lenalidomide: dexamethasone and cyclophosphamide
TGF-β: transforming growth factor-β
VGPR: very good partial response
VEGF: vascular endothelial growth factor.
